# 579 Histopathological Changes in the Endocrine Pancreas of Rats During the Acute Period of Burns

**DOI:** 10.1093/jbcr/irad045.174

**Published:** 2023-05-15

**Authors:** Santiago Santelis, Ayse Ebru Abali, Gonca Ozgun, B Handan Ozdemir, Neslihan Basci Tutuncu, Mehmet Haberal

**Affiliations:** Burn Center and Burn and Fire Disasters Institute, Baskent University, Sanford, Florida; Baskent University, Ankara, Ankara; Baskent University, Ankara, Ankara; Baskent University, Ankara, Ankara; Baskent University, Ankara, Ankara; Baskent University, Ankara, Ankara; Burn Center and Burn and Fire Disasters Institute, Baskent University, Sanford, Florida; Baskent University, Ankara, Ankara; Baskent University, Ankara, Ankara; Baskent University, Ankara, Ankara; Baskent University, Ankara, Ankara; Baskent University, Ankara, Ankara

## Abstract

**Introduction:**

Severe burn victims experience a systemic inflammatory response and a hypermetabolic response. Presence of hyperglycemia, even with concurrent hyperinsulinism, is indicative of insufficient insulin secretion from beta cells to overcome the glycemia. However, the underlying molecular mechanisms of beta-cell failure in patients with acquired insulin resistance after burn trauma are not clearly understood. Our aim in this study was to describe the histopathological changes in the pancreatic islets secondary to severe burns in an experimental animal model.

**Methods:**

Fourteen Wistar albino rats were randomly divided into 2 groups: the sham group and the burn group. A full-thickness burn model was designed to induce a burn of 25% total body surface area. Seven days after burn induction and sham procedure, pancreatectomy was performed. Pancreatic tissues were examined under light microscopy, and islet size and cellularity were calculated.

**Results:**

The histopathologic examination was unremarkable, but the mean number of islets per pancreatic tissue was lower in the burn group than in the sham group. We observed a significant difference in the mean number of cells per one islet between the 2 groups, with the cell count higher in the burn group (*P* < .05).

**Conclusions:**

During the acute phase of burn injury in rats, we observed a decrease in the number of pancreatic islets with remarkable hypercellularity. Further studies are needed to determine the histological and cellular basis of these changes.

**Applicability of Research to Practice:**

Major burn injury causes severe alterations in beta-cell mass, which increases and decreases both function and mass to maintain the glycemic level within a very narrow physiological range. Patients with acute and prolonged hyperglycemia can have complications leading to impaired wound healing, increased skin graft loss, increased muscle protein catabolism, increased incidence of infections, and mortality. Therefore, a detailed understanding of the pancreatic changes under different preexisting conditions and therapeutic interventions in the context of a major burn trauma might guide treatment options.

